# Non-small cell lung cancer with *EML4-ALK* translocation in Chinese male never-smokers is characterized with early-onset

**DOI:** 10.1186/1471-2407-14-834

**Published:** 2014-11-18

**Authors:** Yongjun Guo, Jie Ma, Xiaodong Lyu, Hai Liu, Bing Wei, Jiuzhou Zhao, Shuang Fu, Lu Ding, Jihong Zhang

**Affiliations:** The Affiliated Tumor Hospital of Zhengzhou University, Henan Cancer Hospital, No. 127, Dongming Road, Zhengzhou, Henan 450008 China; The Institute of Forensic Science and Technology of Henan Provincial Public Security Bureau, No. 9 JinShui Road, ZhengZhou, Henan 450003 China; Hematology Laboratory of Hematology Malignancy Treatment Center, Shengjing Hospital of China Medical University, No. 39 Huaxiang Road, Tiexi District, Shenyang, Liaoning 100022 China; Shanghai Yuanqi Bio-pharmaceutical Company Ltd, No. 699 North Huifeng Road, Shanghai, 201403 China

**Keywords:** Non-small-cell lung cancers (NSCLCs), Anaplastic lymphoma kinase (*ALK*), Echinoderm microtubule-associated protein-like 4 (*EML4*), Tyrosine kinase inhibitors (TKIs), Never-smokers, Adenocarcinoma

## Abstract

**Background:**

The translocations of the anaplastic lymphoma kinase (*ALK*) gene with the echinoderm microtubule-associated protein-like 4 (*EML4*) gene on chromosome 2p have been identified in non-small-cell lung cancers (NSCLCs) as oncogenic driver mutations. It has been suggested that *EML4-ALK* fusion is associated with the resistance in NSCLCs to epidermal growth factor receptor tyrosine kinase inhibitors (EGFR TKIs), such as gefitinib and erlotinib. In contrast, ALK tyrosine kinase inhibitor (ALK TKI) crizotinib has shown superior effects in combating NSCLCs with *EML4-ALK*. Thus, characterization of EML4-ALK fusion genes and clinical features of resulting carcinomas would be a great benefit to disease diagnosis and designing customized treatment plans. Studies have suggested that *EML4-ALK* translocation occurs more frequently in never-smokers with NSCLC, especially in female patients. However, it is not clear whether this is the case in male patients, too. In this study, we have determined the frequency of EML4-ALK translocation in male never-smokers with NSCLC in a cohort of Chinese patients. The clinical features associated with *EML4-ALK* translocation were also investigated.

**Methods:**

A cohort of 95 Chinese male never-smokers with NSCLC was enrolled in this study. *EML4-ALK* fusion genes were detected using one-step real time RT-PCR and DNA sequencing. We further determined the expression levels of *ALK* mRNA by RT-PCR and ALK protein by immunohistochemistry in these specimens. The clinical features of *EML4-ALK*–positive carcinomas were also determined.

**Results:**

We have identified *EML4-ALK* fusion genes in 8 out of 95 carcinoma cases, accounting for 8.42% in Chinese male never-smokers with NSCLC. It is significantly higher than that in all Chinese male patients (3.44%) regardless smoking habit. It is also significantly higher than that in all Chinese smokers (8/356 or 2.25%) or in smokers worldwide (2.9%) by comparing to published data. Interestingly, *EML4-ALK* fusion genes are more frequently found in younger patients and associated with less-differentiated carcinomas.

**Conclusions:**

The frequency of *EML4-ALK* translocation is strongly associated with smoking habits in Chinese male patients with higher frequency in male never-smokers. *EML4-ALK* translocation is associated with early-onset and less-differentiated carcinomas.

## Background

Lung cancer is one of the most common cancers and is by far the leading cause of cancer-related deaths worldwide [1]. Nearly 80% of lung cancer patients are diagnosed as non-small cell lung cancer (NSCLC) at advanced stages of the disease [2]. The average survival time is usually short after diagnosis, largely due to unsatisfactory outcomes from conventional chemotherapeutic regimens, especially in patients with metastatic cancer [3]. However, the treatment outcome has been improved significantly in the past several years owing to increased understanding on molecular mechanisms of tumorigenesis. It is highlighted by the finding that the epidermal growth factor receptor tyrosine kinase inhibitors (EGFR TKIs) including gefitinib and erlotinib have shown superior improvement on survival time and life quality in a subset of patients harboring EGFR activating mutations [4, 5]. Thus, current strategies to improve treatment outcomes have been focused on target-specific and customized treatment according to the patient’s molecular profile [6].

The fusion of the anaplastic lymphoma kinase (*ALK*) with the echinoderm microtubule-associated protein-like 4 (*EML4*) on chromosome 2p was first identified as oncogenic driver mutations in 2007 in Japanese NSCLC patients [7]. Out of 75 examined Japanese patients, 5 patients (6.7%) were found carrying the *EML4–ALK* fusion transcript, which resulted from a small inversion within chromosome 2p [7]. Multiple studies have been carried out to determine the frequency of *EML4–ALK* translocation occurrences in patients with NSCLC, ranging from 1.6% to 11.7% in individual studies [7–18] with an averaged frequency at about 5%, estimated from published results [6]. The huge variation among these studies is likely due to the differences in patient selection criteria such as disease status, race, country, gender, and/or smoking habit. Other *ALK-*fusion genes including *KIF5B-ALK* have also been identified in patients with NSCLC [8, 19–21]. It has been suggested that patients with *ALK* rearrangement are resistant to EGFR TKIs [22]. However, crizotinib (XALKORI®, Pfizer Inc.), an ALK tyrosine kinase activity inhibitor, has been approved by the FDA in the United States for treating patients with ALK + advanced NSCLC [23] as well as in other countries, including China.

Although *EML4–ALK* translocation was first identified from a lung adenocarcinoma specimen surgically resected from a 62-years-old man with a history of smoking [7], increased evidence suggests that it is much more common in never-smokers based on the studies performed in different countries [10, 15, 16, 22]. As estimated, the incidence of *EML4-ALK* fusion in never-smokers is 9.4% vs. 2.9% in smokers [6]. In addition to smoking habit, studies also suggest that the frequency of the incidence is different between male and female patients [17, 18]. However, based on the available data from these publications, it is not clear what the frequency is in either male or female never smokers who were diagnosed as NSCLC. A recent study has reported that the incidence could be as high as 15.2% (5/33) in a small cohort of Chinese female adenocarcinoma patients who are never-smokers [18]. However, it is not clear whether the incidence is also high in male never-smokers with NSCLC. To address this question, we assembled 95 Chinese male patients who are never smokers and diagnosed with NSCLC. We used one-step reverse transcription polymerase chain reaction (RT-PCR) to screen *ELM4-ALK* fusion genes in these patients. We have identified 8 (8.42%) cases with *ALK* rearrangement, which is significantly higher than estimated 2.9% in the smokers with NSCLC worldwide [6]. Interestingly, our study suggests that *EML4-ALK* rearrangements in Chinese male never-smokers with NSCLC are more frequently detected in younger patients and in less-differentiated carcinomas.

## Methods

### Patient enrollment and tissue specimens

There are a total of 95 non-smoking Chinese male patients with NSCLC enrolled in this study (Table 1). These patients are from Shengjing Hospital of China Medical University, Hunan Cancer Hospital, Henan Cancer Hospital, China. All participants who underwent surgery provided written informed consent. The study was approved by the Institutional Ethics Committee of Henan Cancer Hospital. Tissue specimens, which were collected from NSCLC patients with suspected NSCLC, were preserved in formalin-fixed paraffin-embedded (FFPE) tissue blocks. These FFPE tissue blocks were subjected to EML4-ALK detection, mRNA and protein level evaluation, and fluorescence in situ hybridization (FISH) analysis. Tumor subtype and pathological characteristics were evaluated independently by two pathologists as a standard procedure during disease diagnosis. In cases with diagnostic disagreement, a third pathologist gave additional independent review. Depending on how closely the cancer cells and tissue resemble normal cells and tissue, tumors were staged using a three-tiered grading system as well differentiated (Grade 1), moderately differentiated (Grade 2), and poorly differentiated (Grade 3). Grade 1 (low grade) tumors appear close to normal and tend to grow and spread slowly. Grade 2 and 3 tumors look abnormal and tend to grow more rapidly and spread faster than tumors with a lower grade. Collectively, Grade 2 and 3 tumors are described as less-differentiated carcinomas.

**Table 1:** **Clinical characteristics of 95 Chinese male never-smokers with NSCLC**

Characteristics		No. (patients)	% (patients)
Age (years)	<40	1	1.05%
	40-49	20	21.05%
	50-59	27	28.42%
	≥60	47	49.47%
Differentiation	poor	31	32.63%
	moderate	39	41.05%
	well	25	26.32%
Histology	Adenocarcinoma	84	88.42%
	Squamous	6	6.32%
	Others	5	5.26%

### Identification of EML4-ALK fusion gene

FFPE tissue blocks were sectioned onto slides for hematoxylin and eosin (H&E) staining. The sections for further study were left unstained. Tumor areas were identified and collected for RNA extraction as described below. Total RNA was extracted with RNeasy FFPE Kit (Qiagen, CA, USA) following the manufacturer’s instructions. Extracted RNA samples were treated with DNase I (DNA-*free*; Applied Biosystems-Ambion, TX, USA) to remove any DNA contamination before one-step RT-PCR. These RNA samples were then subjected to one-step RT-PCR to detect *EML4-ALK* fusion transcripts using human Lung Cancer Related Fusion Gene Detection Kit (fluorescence RT-PCR) (Shanghai Yuanqi Bio-Pharmaceutical Co., Ltd.). The sequences of the PCR primers as well as DNA sequencing primers are shown in Table 2. RT-PCR was also performed using a different set of primers that were previously published [24]. The mixture of each reaction contains 3 μL total RNA, 20 μL Multiplex RT-PCR Buffer, 2 μL Multiplex Enzyme Mix in a total volume of 25 μL. The RT-PCR was performed at 42°C for 30 min, at 94°C for 5 min followed by 40 cycles 94°C for 15 s and 60°C for 1 min on the 7300 Real Time PCR System (ABI, USA). All positive cases identified by RT-PCR were confirmed by both DNA sequencing and Vysis ALK Break Apart FISH analysis.

**Table 2:** **Sequences of primers used for detection of subtypes of EML4-ALK fusion**

Forward primers		
Subtypes of fusion	Location	Sequences
E2-A20 (V5a), E2-ins117-A20 (V5b)	EML4-E2	GCTAAAGGCGGCTTTGGCTG
E6-A20 (V3a), E6b-A20 (V3b)	EML4-E6	AGTCACATAATTCTTGGGAA
E13-A20 (V1), E13-ins49-A20 (V6)	EML4-E13	ATTTGTGCAGTGTTTAGCATTC
E14-ins11-A20 (V4b), E14-del12-A20 (V7)	EML4-E14	GGGAAAGGACCTAAAGGTG
E15-A20 (V4a)	EML4-E15	GTAGCAGAAGGAAAGGCAGATC
E17-A20, E18-A20 (V9)	EML4-E17	CGCTACTCAATAGATGGTACCT
E20-A20 (V2)	EML4-E20	CGGGAGACTATGAAATATTGTACT
Common reverse primer	ALK-E20	CATGATGGTCGAGGTGCG C
Sequencing primer		TTGCTCAGCTTGTACTCAGGGCTCTG

### ALK tyrosine kinase expression analysis

Primers specific to ALK tyrosine kinase domain (ALK TK) were designed for ALK mRNA expression analysis. The expression level of housekeeping gene ABL was also determined as the control. The primers used for ALK and ABL are: ALK-forward 5′-AGAAACTGCCTCTTGACCTG-3′; ALK-reverse 5′-GGGCATCCACTTAACTGGC-3′, ABL-forward 5′-TACCTGAGGGAGTGCAACC-3′, ABL-reverse 5′-TTTTCTTCTCCAGGTACTCCA-3′. The DNA sequencing primers include ALK-sequencing primer 5′-CCCTTTCTATAGTAGCTCGCCCTGTAGAT-3′ and ABL-sequencing primer 5′-CCATGTACAGCAGCACCACGGCGT-3′.

The expression of ALK tyrosine kinase was examined by immunohistochemistry using rabbit monoclonal antibody against ALK (clone D5F3, Cell Signaling Technology, Inc., USA), which detects endogenous levels of total ALK protein as well as ALK fusion proteins. The experiments were performed on FFPE sections, as described in detail previously [25]. In brief, after deparaffinization, tissue sections were incubated with 3% H2O2 to block endogenous peroxidase activity. Heat-induced antigen retrieval was then performed for 30-60 minutes using the Ventana CC1 mild reagent containing a combination of ethylenediaminetetraacetic acid (EDTA) and boric acid in Tris buffer (Ventana Medical Systems, Inc., USA). After blocking with 10% normal goat serum, the abovementioned rabbit monoclonal antibody against ALK (D5F3) was applied, followed by incubation with horseradish peroxidase-conjugated multimer antibody (Ventana Medical Systems). The antigen-antibody complex was visualized using diaminobenzidine (UltraView, Ventana Medical Systems) and the slides were slightly counterstained with hematoxylin. Slides were evaluated for intensity (0 no staining; 1+ light staining; 2+ moderate staining; 3+ strong staining) and distribution of ALK immunostaining. Any IHC intensity greater than 0 was defined as IHC positive.

### ALK Break Apart FISH analysis detecting *ALK*rearrangement

To identify ALK rearrangements, FISH experiments were performed on FFPE tissue sections by using Vysis ALK Break Apart FISH Probe Kit (Abbott Molecular Inc., USA) according to vendor’s instruction. In brief, DNA probes that flank the *ALK* breaking point and are labeled with green and red florescent dye separately were used in FISH on tumor sections. When hybridized against normal nuclei (negative cells), the fluorescent signals (red and green fluorescent dots) from two probes are colocalized (merged as a yellowish signal) or less than two signal diameters apart. However, when *ALK* rearrangement occurs, the signals from two probes are more than two signal diameters apart in a single nucleus (positive cell). A sample is considered negative if <5 cells out of 50 (<5/50 or <10%) are positive, positive if >25 cells out of 50 (>25/50 or >50%) are positive, or equivocal if 5 to 25 cells (10 to 50%) are positive, which needs a further evaluation (See vendor’s product manual for more details).

## Results

### Characterization of enrolled patients

To determine the frequency of *EML4-ALK* rearrangement in a cohort of NSCLC patients who are male never-smokers in a single ethnic group, we assembled 95 Chinese male never-smoker patients who were diagnosed as NSCLC from January 2012 to June 2013. The major clinical features of these patients are summarized in Table 1. The median age of these patients is 58.76 years old (yrs), ranging from 38 to 76. Nearly half of the patients are at their 60s or older, while 28.42% of the patients are in their fifties and 21.05% in their forties. The majority of the patients are diagnosed as adenocarcinoma (84/95 or 88.4%). Only 6.32% of the patients carry squamous carcinomas and 5.26% carry other types of carcinomas. These patients can be divided into three subgroups based on the differentiation levels of their carcinomas cells. The number of patients is not significantly different among three groups with 31, 39, or 25 patients in each group carrying poorly, moderately, or well differentiated carcinomas, respectively.

### Detection and characterization of EML4-ALK rearrangements

**Figure 1:**
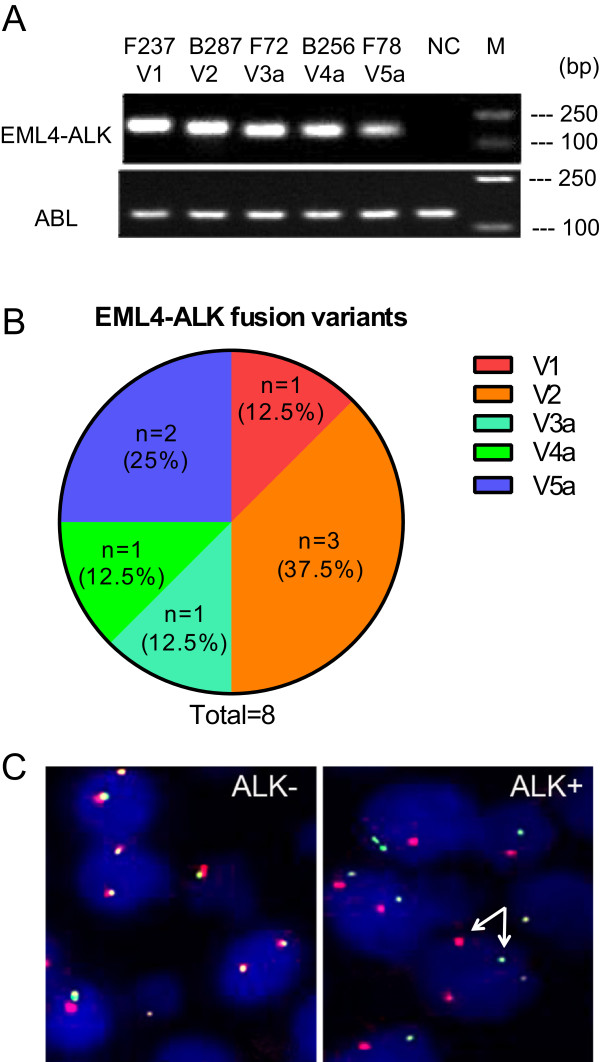
**Screening for EML4-ALK fusion genes in Chinese male never-smokers with NSCLC. (A)** DNA gel electrophoresis showing the expression of *EML4-ALK* fusion genes (top panel) and ABL controls (bottom panel) by one-step real time RT-PCR in Chinese male never-smokers. One sample is shown for each detected fusion variant (V1, V2, V3a, V4a, and V5a) as indicated below the case numbers. NC, negative case; M, DNA ladder. **(B)** The frequency of different *EML4-ALK* variants. **(C)** ALK Break Apart FISH analysis showing *ALK* inversion in an *EML4-ALK*-negative (ALK-, left) and –positive (ALK+, right) carcinoma tissue. The inversion is indicated by two separated red and green fluorescent dots that are used to label two probes targeting the sequences flanking the breaking point. One such example is indicated by a pair of arrows in ALK + image.

**Figure 2:**
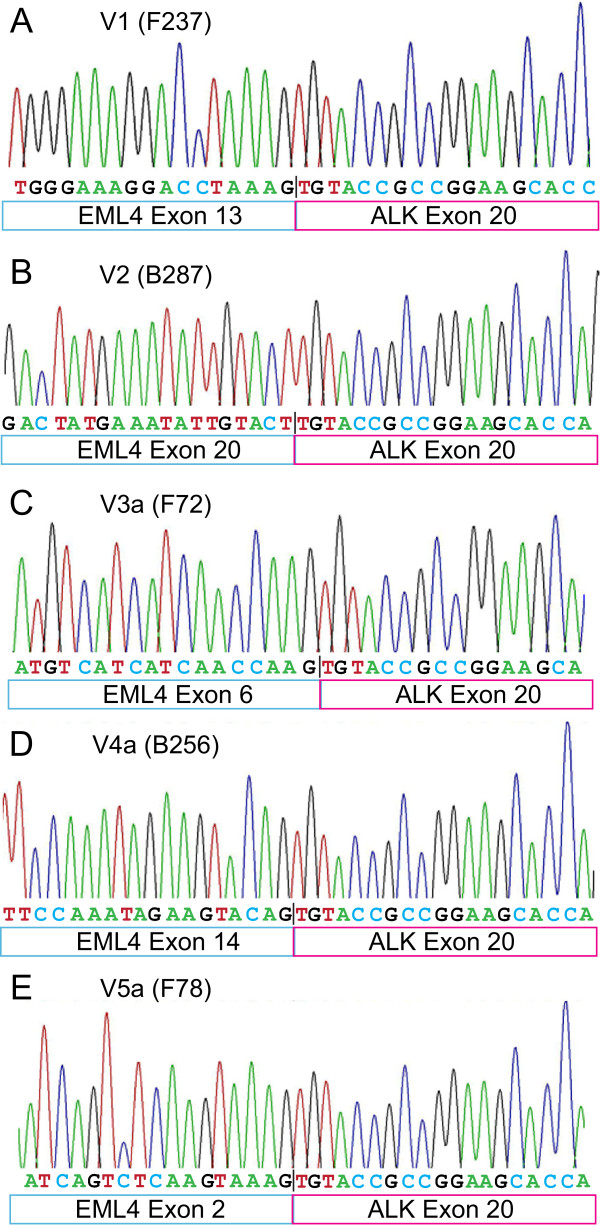
**Representative DNA sequencing chromatographs and schematic drawings showing** ***EML4-ALK*** **fusion junctions.** Five *EML4-ALK* fusion transcript variants were detected by one-step real time RT-PCR. The DNA sequences and their sequencing traces are shown for each detected fusion transcripts: V1 **(A)**, V2 **(B)**, V3a **(C)**, V4a **(D)**, and V5a **(E)**. The Case ID is also indicated for each sample. Schematic drawings showing the organization of fusion junctions are shown below the corresponding DNA sequences.

FFPE samples were prepared from resected lung carcinomas of enrolled patients. To detect *EML4-ALK* rearrangement, we extracted total RNAs from FFPE samples and performed one-step real time RT-PCRs using primers that recognize all known *EML4-ALK* fusion transcript variants. By this method, we detected *EML4-ALK* translocation in 8 carcinomas, accounting for 8.42% in 95 Chinese male never-smokers with NSCLC. We have detected a total of five different types of *EML4-ALK* fusion transcript variants, as shown in the DNA gel image (Figure 1A). These results were verified by additional RT-PCR experiments using a set of previously published primers [24]. Among 8 *EML4-ALK* positive carcinomas, there are three cases (37.5%) with variant 2 (V2), two cases (25%) with V5a and one each (12.5%) with variant V1, V3a, or V4a, respectively (Figure 1B). The frequency of the incidence for each variant is 1.05% for V1, V3a, or V4a, 3.16% for V2, and 2.11% for V5a in Chinese male never-smokers with NSCLC (Figure 1B). *ALK* rearrangements were confirmed by using the Vysis ALK Break Apart FISH Probe Kit, which detects *ALK* rearrangements using two fluorescent-labeled probes flanking the *ALK* breaking point. As shown in Figure 1C, *ALK* rearrangements, indicated by two separated fluorescent signals (for example, orange and green signals indicated by a pair of arrows in the right panel) in a distance at least two signal diameters apart in a single nucleus, are detected in fusion-positive carcinoma (*ALK*+, right panel), but not (or less frequently in some cases) in fusion-negative carcinoma (*ALK*-, right panel). We further confirmed the identity of these RT-PCR products by DNA sequencing. The DNA sequencing traces are shown in Figure 2. The cartoons showing the organization of exons from EML4 and ALK for each variant are also included in the above mentioned figure.

### ALK mRNA and protein are exclusively expressed in *EML4-ALK*-positive carcinomas

**Figure 3:**
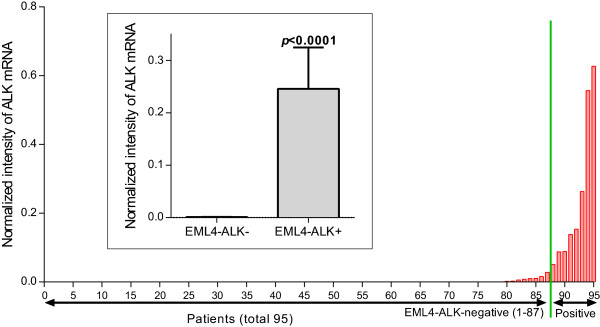
**Expression of ALK mRNA in Chinese male never-smokers with NSCLC.** The expression level of ALK mRNA in each carcinoma (total 95 specimens) was determined by one-step real time RT-PCR using primers specific to the ALK TK domain and presented as the intensity normalized to the housekeeping gene ABL. While ALK mRNA is not expressed or expressed at very low levels in *EML4-ALK* negative carcinomas, it is aberrantly expressed in all *EML4-ALK* positive samples. The mean intensities are displayed in the inserted bar graph for both *EML4-ALK* negative or positive patients, showing a significant difference in the expression levels of the two groups. Values are means ± SEM in the insert. *p* value was determined by student t test.

**Figure 4:**
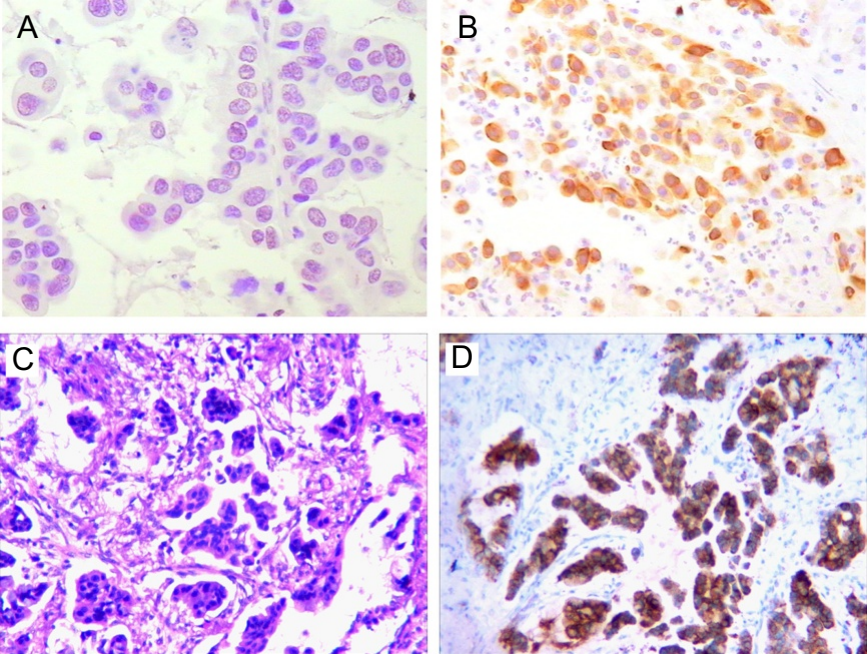
**Representative images showing the association between ALK protein expression and carcinoma differentiation.** H&E staining and immunohistochemistry using an antibody against ALK were performed on the same FFPE tissue section from an *EML4-ALK*-negative **(A)** or an *EML4-ALK*-positive **(B)** specimen. **(A)** Representative image showing the absence of any ALK protein in a well differentiated, *EML4-ALK*-negative carcinoma. **(B)** Representative image showing the aberrant expression of ALK protein in a poorly differentiated, *EML4-ALK*-positive carcinoma. **(C)** Representative image showing histopathology of mucinous cribriform carcinoma. **(D)** Representative image showing ALK immunostaining in a section adjacent to the one shown in panel **(C)**.

One-step real-time RT-PCRs were performed to measure the expression level of ALK in all 95 samples using primers specific to ALK TK domains, which are included in the mRNA transcribed from both intact *ALK* genes and *EML4-ALK* fusion genes. We have found that ALK TK mRNA level (normalized to house gene ABL) in the *EML4-ALK*-positive carcinomas is significantly higher (*p* < 0.0001 by Mann–Whitney test) than in the *EML4-ALK*-negative samples (Figure 3). As shown in the scatter plot, the expression level of ALK TK mRNA is either non-detectable or at very low level. This result suggests that the expression of ALK mRNAs is the result of ALK translocation and that ALK mRNAs are mostly transcribed from the *EML4-ALK* fusion gene, but not from the intact *ALK* gene. We also performed immunohistochemistry using anti-ALK antibody and found that ALK protein is highly expressed in *EML4-ALK* positive samples, but not (or at very low level) in *EML4-ALK* negative samples (see Figure 4A-B for representative images). As reported previously [9, 26, 27], we found a mucinous cribriform pattern in two of *EML4-ALK*-positive adenocarcinomas (Figure 4C-D). Together, these results suggest that *EML4-ALK* rearrangement results in aberrant expression of *ALK* TK domain at both mRNA and protein levels. These results are also consistent with the notion that *EML4-ALK* is an oncogene, whose abnormal ALK tyrosine kinase activity is likely responsible for the tumorigenesis in the patients with *ALK* translocation.

### Clinical characterization of EML4-ALK positive patients

Our results suggest that *ALK* rearrangements are usually detected in younger patients. The median age of *EML4-ALK* positive patients is 50.63 yrs vs. 58.88 yrs in all patients. It ranges from 40 to 69 yrs, with 5 of them at their 40s, one at 52 yrs, and two at their 60s. As listed in Table 3, the difference in the ages between patients with and without *EML4-ALK* is significant (*p* < 0.02 by Chi-square test), suggesting an early-onset of NSCLC in *EML4-ALK* positive patients. Among all 8 *EML4-ALK* positive patients, two were diagnosed as squamous carcinomas (Table 4), with one at the age of 46 and one at the age of 67. Therefore, it is hard to conclude with this small sample size whether there is any age preference in the disease onset in the patients with squamous carcinomas. In the patients with adenocarcinoma, disease onset predominantly occurs in younger patients, with all but one (5 vs. 1) at the age of 52 yrs and younger. Comparing to the observation that 26 patients are 52 yrs or younger and that 63 patients are older than 52 yrs in all the patients with adenocarcinomas, the difference is significant (*p* < 0.013, by Fisher’s exact test). This result suggests that NSCLC onset age in Chinese male never-smokers is lower in *EML4-ALK*–positive patients, especially in those diagnosed as adenocarcinomas.

**Table 3:** **Distribution of EML4-ALK fusions in different age groups**

Age (years)	EML4-ALK+	EML4-ALK-
<40	0	1
40-49	5	15
50-59	1	26
≥60	2	45

*p* < 0.02 by Chi-square test.

**Table 4:** **Clinical features of EML4-ALK positive carcinomas in Chinese male never-smokers with NSCLC**

Patients	Histology	Differentiation	Variant	Size	EML4	ALK
F25	adenocarcinoma	poor	V2	177 bp	E20	A20
F78	adenocarcinoma	poor	V5a	155 bp	E2	A20
F133	adenocarcinoma	poor*	V2	177 bp	E20	A20
F237	adenocarcinoma	poor*	V1	185 bp	E13	A20
B256	adenocarcinoma	moderate	V4a	162 bp	E15	A20
B287	squamous	moderate	V2	177 bp	E20	A20
B375	squamous	moderate	V5a	155 bp	E2	A20
F72	adenocarcinoma	well	V3a	162 bp	E6	A20

*carcinomas with cribriform patterns.

We next examined the differentiation levels of carcinomas in *EML4-ALK* positive carcinomas. All carcinomas except one are less-differentiated, including 4 poorly and 3 moderately differentiated cases (Table 4), suggesting that *EML4-ALK* rearrangements usually occurred in less-differentiated carcinomas. The 7 less-differentiated carcinomas include two moderately differentiated squamous carcinomas, four poorly differentiated adenocarcinomas, and one moderately differentiated adenocarcinoma. The only well differentiated carcinoma is diagnosed as adenocarcinoma. Taken together, these results suggest that *EML4-ALK* translocation occurs more frequently in less-differentiated carcinomas with an early-onset in Chinese male never-smokers with NSCLC.

### Analysis of EML4-ALK in Chinese NSCLC patients

We performed an analysis using the data extracted from 6 published studies [8, 14, 16–18, 28] that were carried out exclusively in Chinese patients with NSCLC (Table 5). We choose to use these data to avoid bias that may be caused by genetic (race) and environmental (country) differences while comparing to our analysis. By this analysis, the average frequency of *EML4-ALK* translocation in non-selected Chinese patients [8, 14, 16–18] is 5.50% (43/782) (Table 5), which is similar to the values estimated from the patients worldwide. This analysis further indicates that the frequency in Chinese never-smokers (35/375 or 9.33%) is also significantly higher (*p* < 0.0001 by Fisher’s exact) than in Chinese smokers (8/356 or 2.25%). By comparing to our results, the frequency of *EML4-ALK* translocation is significantly higher (*p* < 0.01 by Fisher’s exact) in Chinese male never-smokers with NSCLC (8/95 or 8.42%) than in all Chinese smokers with NSCLC.

**Table 5:** **Frequency of EML4-ALK in Chinese NSCLC Patients (extracted from 6 published studies)**

Publication [ref]	All Patients		Never-smokers		Smokers		Female		Male	
	Total	EML4-ALK	Total	EML4-ALK	Total	EML4-ALK	Total	EML4-ALK	Total	EML4-ALK
	No.	No. (%)	No.	No. (%)	No.	No. (%)	No.	No. (%)	No.	No. (%)
Rekova et al. 2007 [8]	103	3 (2.91%)								
Wong et al. 2009 [14]	266	13 (4.89%)	141	10 (7.09%)	125	3 (7.09%)	134	8 (5.97%)	132	5 (3.79%)
Zhang et al. 2010 [16]	103	12 (11.65%)	52	10 (19.23%)	51	2 (19.23%)	29	5 (17.24%)	74	7 (9.46%)
Shaozhang et al. 2012 [17]	102	8 (7.84%)	52	6 (11.54%)	50	2 (11.54%)	48	6 (12.50%)	54	2 (3.70%)
Li et al. 2013 [18]	208	7 (3.37%)	78	6 (7.69%)	130	1 (7.69%)	61	7 (11.48%)	147	0 (0.00%)
Sun et al. 2010 [28]			52	3 (5.77%)						
Sum	782	43 (5.5%)	375	35 (9.33%)	356	8 (2.25%)	272	26 (9.56%)	407	14 (3.44%)

By Fisher’s exact test, the difference is significant between (i) never-smokers and smokers (*p* < 0.0001); (ii) Female and Male (*p* < 0.002).

We next calculated the frequency of *EML4-ALK* translocation in male and female Chinese patients with NSCLC regardless of smoking history (Table 5). It is significantly higher (*p* < 0.002 by Fisher’s exact) in Chinese female patients (26/272 or 9.56%) than in Chinese male patients (14/407 or 3.44%). By comparing this to our results, the incidence of EML4-ALK translocation in Chinese male patients with NSCLC is significantly higher (*p <* 0.048 by one-tailed Fisher’s exact) in never-smokers (8/95 or 8.42%) than in all male patients regardless of smoking history (14/407 or 3.44%).

Taken together, we have found that both smoking habit and gender are associated with the frequency of *EML4-ALK* translocation in Chinese NSCLC patients. It is more frequently detected in female than in male patients and more in never-smokers than in smokers. In Chinese male patients with NSCLC, *EML4-ALK* translocation is also more frequently detected in never-smokers than in smokers.

## Discussion

In this study, we have determined the frequency of *EML4-ALK* translocation and its associated clinical features in Chinese male never smokers with NSCLC. A previous study has shown that the frequency of *EML4-ALK* translocation in Chinese female patients with NSCLC is higher in never-smokers. Similar to that, we have found that the incidence in Chinese male NSCLC patients is also significantly higher in never-smokers by comparing to published data. Interestingly, we have also found that these Chinese male never-smoker patients in our study are strongly associated with younger-onset and less-differentiated carcinomas, which are likely caused by aberrant expression of ALK mRNA and protein.

Since *EML4-ALK* fusion genes were first identified as potential driver mutations in NSCLC [7], multiple studies have been carried out to determine the frequency of *EML4-ALK* translocation. The frequencies of *EML4-ALK* may vary substantially among different publications, ranging from 1.6% in a cohort of Japanese patients [15] to 11.7% in a cohort of Chinese patients [16]. The large variation is likely contributed by multiple factors, including race, gender, smoking habit, etc. Several groups have estimated the frequency of *EML4-ALK* translocation worldwide by combining published data and found the value at 5% [6], 3.7% [17], or 4.8% [18], respectively. The differences among these values are caused by differences in the selection of data sources. To exclude the influence caused by differences in races (genetic factors) and countries (geographic, cultural, environmental, diet, etc.), and also to better determine the influence of gender and smoking habit in the occurrence of *EML4-ALK* translocation, we performed an analysis using the data extracted from published studies that were done exclusively in Chinese patients (Table 5) [8, 14, 16–18]. The average frequency of *EML4-ALK* in non-selected Chinese patients is 5.50% (Table 5), which is similar to the estimated number using published studies in the patients from multiple countries. This result suggests that race and country have the least effect in the occurrence of *EML4-ALK* translocation in NSCLC. Interestingly, our analysis has shown that both gender and smoking habit are important factors affecting the frequency of *EML4-ALK* translocation in Chinese patients. *EML4-ALK* translocation is more frequent in Chinese never-smokers than in Chinese smokers, which is consistent with the result in patients worldwide [6]. Our analysis has also clearly shown that the difference between Chinese male and Chinese female patients is highly significant (*p* < 0.002, Table 5).

Interestingly, a previous study has found that the frequency of *EML4-ALK* translocation is as high as 15.2% (5/33) in Chinese female non-smokers with NSCLC [18]. Although double the amount, it is not significantly different (by Fisher’s exact) when compared to the frequency in Chinese male never-smokers from our study (8.42% or 8/95). Therefore, more patients are needed to draw a solid conclusion on whether there is a significant difference in the frequency of *EML4-ALK* translocation between male and female never-smokers. In addition, authors in this study detected zero patients with *EML4-ALK* translocation in 147 Chinese male patients, including 45 never-smokers (calculated from available data in the paper) [18]. The failure to detect any positive cases in male patients is likely due to the small sample size and low frequency (3.44%, Table 5) in Chinese male patients with NSCLC. As determined in our current study, the frequency in Chinese male never-smokers with NSCLC (8.42%) is significantly higher than that in all Chinese male patients with NSCLC (2.25%, Table 5). It should be noted that although we enrolled consecutive patients who met our criteria, our samples are predominantly collected from adenocarcinomas (88.4%), which may bias our results and interpretations.

We have detected 5 different types of *EML4-ALK* variants (V1, V2, V3a, V4a, and V5a). It was the first time that V4 was detected in Chinese NSCLC patients based on previously published results [8, 14, 16–18]. Both ALK mRNAs and proteins are highly expressed in these *EML4-ALK* positive carcinomas (Figures 3 and 4), which is consistent with the notion that *EML4-ALK* rearrangements cause aberrant expression of *EML4-ALK* fusion oncogene and overactivation of ALK tyrosine kinase, which in turn leads to the inhibition of apoptosis and promotion of tumor cell proliferation [25, 29, 30]. Crizotinib, a selective inhibitor of *ALK* and mesenchymal epithelial growth factor tyrosine kinases, has shown significant improvement in response rates and response duration in *ALK*-positive patients in clinical trials [25]. Meanwhile, it has been shown that *ALK*-positive patients are resistant to EGFR TKIs [22]. It is then possible that nearly one-tenth of male never-smokers with NSCLC (8.42%) would likely respond to crizotinib, but not to EGFR TKIs (gefitinib and erlotinib) or drugs targeting other tyrosine kinases. Therefore, at least among Chinese patients, identification of *EML4-ALK* translocation is crucial for predicting the resistance to EGFR TKIs and the responsiveness to ALK TKIs. Ultimately, it will facilitate the planning of an effective treatment and improve the outcome of such treatment in NSCLC.

In this study, we have identified clinical features that are associated with *EML4-ALK* translocation in Chinese male never-smokers with NSCLC. We have found that the median age in these patients (50.63 yrs) is significantly younger than in all studied patients (58.76 yrs). Thus, *EML4-ALK* translocation in Chinese male never-smokers is more likely to occur in younger patients. In other words, the disease onset is much earlier in *EML4-ALK*-positive patients in a subset of Chinese NSCLC patients who are male never-smokers. A previous study has reported that the *EML4-ALK*-positive lung adenocarcinomas are characterized as less-differentiated [31]. In that report, the differentiation levels were determined in 11 *EML4-ALK* positive carcinomas, which were identified by the same group previously [32]. While only one patient carried the carcinomas that were well-differentiated, 10 other patients carried tumors that were poorly or moderately differentiated. In line with this result, our study has shown that *EML4-ALK* rearrangements are predominantly found in carcinomas with less (poorly or moderately) differentiated cells, with the exception of one with well differentiated cells (Table 4). However, there is a difference between the two studies. While all patients in our study are never-smokers, the 11 *EML4-ALK* positive patients in other study include 6 never-smokers and 5 smokers. Thus, it is possible that the differentiation of carcinomas, regardless of smoking history, is associated with the expression of *EML4-ALK* mutant genes. In other words, the aberrant activity of ALK tyrosine kinase induced by *EML4-ALK* translocation results in less-differentiated carcinomas, more dangerous and aggressive types of cancers.

## Conclusions

In summary, the frequency of *EML4-ALK* translocation in Chinese male never-smokers with NSCLC is 8.42%, which is significantly higher than that in all Chinese male patients or Chinese smokers. This result suggests that *EML4-ALK* translocation in Chinese male NSCLC patients is associated with smoking habits. In this subset of NSCLC patients who are Chinese male never smokers, *EML4-ALK* translocation is associated with early-onset and less-differentiated carcinomas, which are likely caused by the aberrant expression of ALK mRNA and protein. These results will significantly enhance our understanding about NSCLC and facilitate disease diagnosis and designing customized treatment plans, thus leading to the improvement in the survival time and life quality in the patients with *EML4-ALK* translocation.

## Abbreviations

ALK: The anaplastic lymphoma kinase
EML4: The echinoderm microtubule-associated protein-like 4
NSCLCs: Non-small-cell lung cancers
EGFR: Epidermal growth factor receptor
TKI: Tyrosine kinase inhibitor
RT-PCR: Reverse transcription polymerase chain reaction
FFPE: Formalin-fixed paraffin-embedded
FISH: Fluorescence in situ hybridization.
